# IgA Vasculitis Presenting As Isolated Monoarthritis Mimicking Transient Synovitis: A Diagnostic Challenge With Delayed Purpura

**DOI:** 10.7759/cureus.109001

**Published:** 2026-05-16

**Authors:** Saad B Sadiq, Vidhu Ashok, Abdulhamid Y Saidu

**Affiliations:** 1 General Practice, Lifeline Hospital, Salalah, OMN; 2 Pediatrics, Lifeline Hospital, Salalah, OMN; 3 Child Health, Nigerian Institute of Medical Research, Lagos, NGA; 4 Community Medicine, Yobe State University, Damaturu, NGA

**Keywords:** diagnostic challenge, henoch-schönlein purpura, iga-mediated vasculitis, mesenteric lymphadenitis, monoarthritis, nonspecific abdominal pain, oligoarthritis, palpable purpura, pediatric vasculitis, transient synovitis

## Abstract

IgA vasculitis (IgAV), previously known as Henoch-Schönlein purpura, is a small-vessel vasculitis that may involve the skin, joints, gastrointestinal tract, and kidneys. While purpura is a key clinical feature, presentations without rash at onset can occur and may lead to diagnostic uncertainty.

We report the case of a two-year-old boy who presented with a three-day history of acute right knee pain, swelling, and reduced weight-bearing ability following a recent febrile respiratory illness. Ultrasound of the knee demonstrated mild periarticular fluid, and a provisional diagnosis of transient synovitis was made. No rash or systemic features were present at initial presentation.

Two days later, the child developed a progressive rash over the lower limbs with erythematous lesions and evolving purpuric features, along with right elbow swelling and intermittent abdominal pain. These findings prompted further evaluation and led to a clinicoradiological diagnosis of IgAV. Abdominal ultrasound excluded intussusception and demonstrated mesenteric lymphadenitis.

Laboratory investigations showed mild inflammatory changes and reactive thrombocytosis, while renal function and serial urinalysis remained normal. The patient was managed conservatively with close monitoring and safety-netting, without corticosteroid therapy. Symptoms gradually resolved, and no renal involvement was identified during follow-up. This case highlights an atypical presentation of IgAV in which isolated monoarthritis preceded the development of purpura, emphasizing the importance of diagnostic vigilance and serial clinical reassessment.

## Introduction

IgA vasculitis (IgAV), previously known as Henoch-Schönlein purpura, is the most common small-vessel vasculitis in children [1,2]. It is characterized by IgA-mediated immune complex deposition affecting small vessels, with involvement of the skin, joints, gastrointestinal tract, and kidneys [1,2]. The classical presentation includes palpable purpura, arthritis or arthralgia, abdominal pain, and renal involvement, with the rash typically serving as the defining diagnostic feature [1-3].

IgAV predominantly affects children between the ages of 3 and 15 years and is often preceded by an upper respiratory tract infection, suggesting an immunologically triggered process [2,3]. The pathophysiology involves IgA1-dominant immune complex deposition within vessel walls, leading to leukocytoclastic vasculitis and downstream inflammatory effects [1,2].

Although diagnosis is often straightforward once purpura develops, atypical presentations are increasingly recognized, particularly in younger children, in whom noncutaneous manifestations may precede the rash [2-4]. This temporal dissociation may lead to diagnostic uncertainty and misclassification as more common pediatric conditions, including transient synovitis, septic arthritis, or reactive arthritis [2-4].

Recognition of such evolving presentations is critical, as IgAV is a dynamic disease requiring ongoing clinical reassessment rather than reliance on initial findings alone [2-4]. We report a case in which isolated monoarthritis initially led to a provisional diagnosis of transient synovitis, with IgAV becoming apparent as additional cutaneous and gastrointestinal features evolved over time.

## Case presentation

A two-year-old boy presented with a three-day history of right knee pain, swelling, and reduced ability to bear weight. This was preceded by a febrile illness with lower respiratory symptoms one week earlier, which had resolved following treatment with amoxicillin-clavulanic acid. The patient had no known drug allergies, including to penicillin. There was no history of trauma.

On examination, the child was afebrile, active, and playful. The right knee was mildly swollen with reduced range of motion due to pain. No rash or other systemic abnormalities were present at the time of initial evaluation.

Ultrasound of the right knee demonstrated mild periarticular fluid with subcutaneous edema, without evidence of joint effusion or collection. A provisional diagnosis of transient synovitis was made, and the patient was managed conservatively with analgesia and observation.

**Figure 1:**
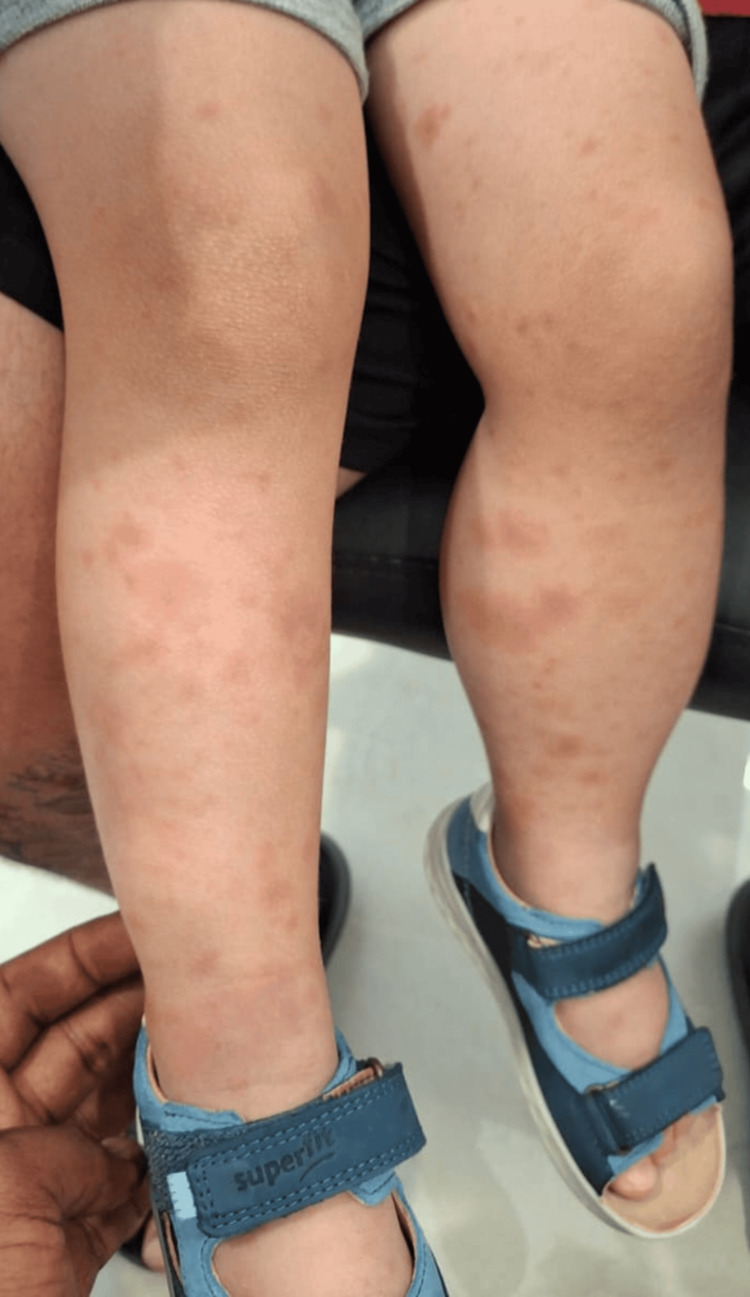
Early cutaneous phase of IgA vasculitis demonstrating erythematous maculopapular lesions predominantly over the extensor surfaces of the lower limbs, with subtle areas suggestive of early evolving purpuric change

Over the subsequent days, the clinical course evolved. Two days later (day 5 of illness), the child developed a rash over the lower limbs, presenting as erythematous maculopapular lesions with subtle areas suggestive of early evolving purpuric change (Figure 1), along with new-onset swelling and pain of the right elbow. Concurrently, the child developed intermittent abdominal pain without vomiting, gastrointestinal bleeding, or abdominal distension, prompting further evaluation. Abdominal ultrasound performed on the same day excluded intussusception and demonstrated mesenteric lymphadenitis.

**Figure 2:**
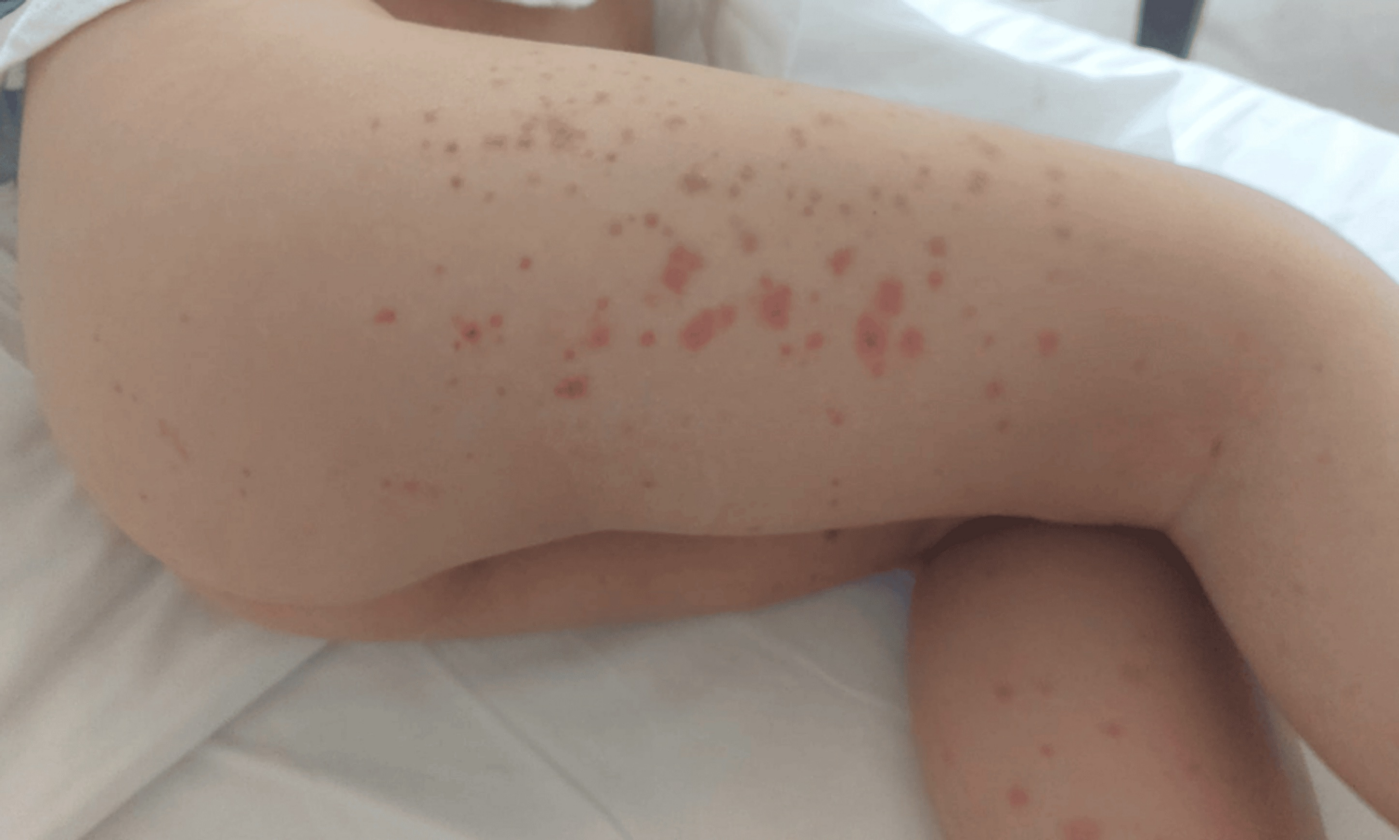
Progressive cutaneous involvement demonstrating increasing lesion density with partial coalescence of erythematous maculopapular lesions over the thigh

**Figure 3:**
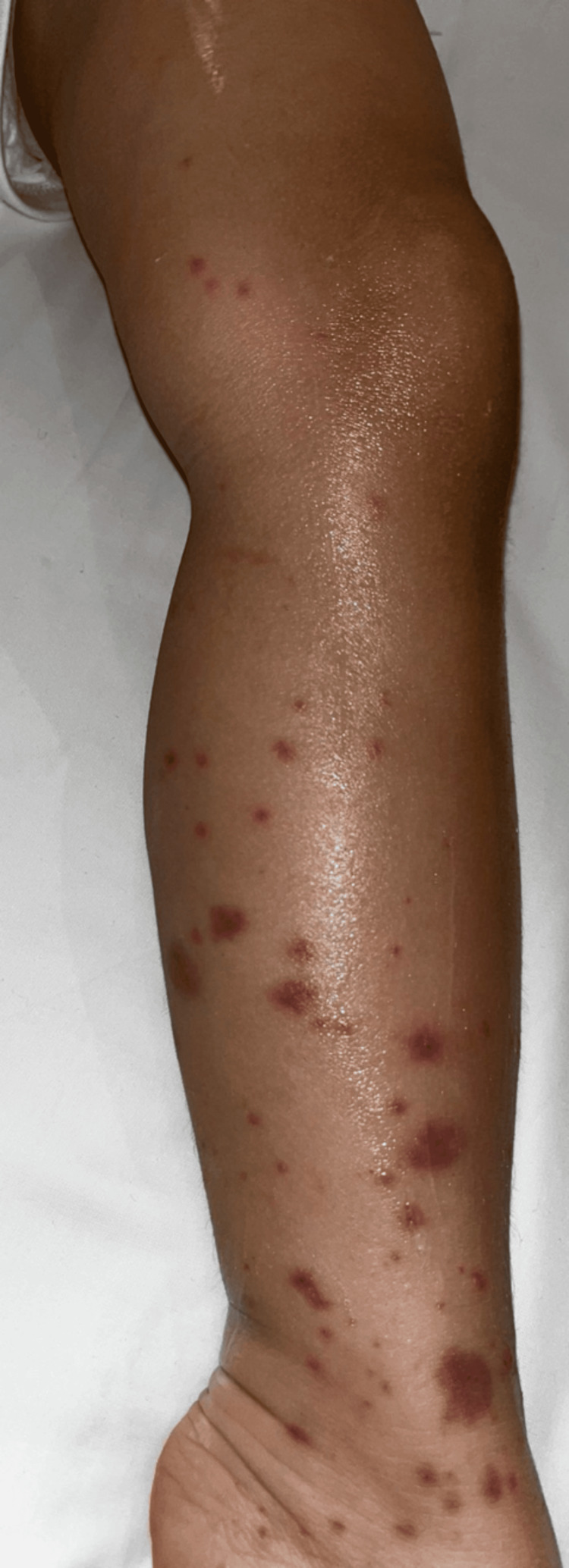
Established purpuric phase demonstrating multiple nonblanching purpuric lesions of varying size, with areas of confluence, over the lower extremity

In the context of preceding monoarthritis, cutaneous involvement, and gastrointestinal symptoms, a clinicoradiological diagnosis of IgAV was made on day 5 of illness. The rash subsequently evolved over the following days, with increasing lesion density and partial coalescence noted on day 6 (Figure 2), and progression to an established purpuric phase characterized by nonblanching palpable lesions over the lower limbs by day 7 (Figure 3).

The child remained systemically stable. Laboratory findings are summarized in Table 1. Initial investigations demonstrated mild leukocytosis, reactive thrombocytosis, and mildly elevated inflammatory markers. Renal function remained preserved, and serial urinalysis showed no hematuria or proteinuria.

**Table 1:** Laboratory findings at presentation and serial urinalysis during follow-up ESR: erythrocyte sedimentation rate; CRP: C-reactive protein

Test (unit)	Observed value	Normal range
Hemoglobin (g/dL)	11.4	11-14
White blood cells (×10⁹/L)	11.5	4-11
Platelets (×10⁹/L)	756	150-450
ESR (mm/hour)	18	<20
CRP (mg/L)	5.18	<5
Blood urea (mg/dL)	37.17	15-45
Serum creatinine (mg/dL)	0.36	0.2-0.5
Uric acid (mg/dL)	3.78	2.4-7.0
Urinalysis (protein)	Negative (serial)	Negative
Urinalysis (hematuria)	Negative (serial)	Negative

The patient was managed conservatively with supportive care. Corticosteroids were not initiated, as symptoms were mild and self-limiting. Caregivers were counseled regarding warning signs and the need for ongoing monitoring.

The chronological progression of clinical features, examination findings, investigations, and management decisions is summarized in Table 2, highlighting the evolving nature of the disease and the transition from an initial diagnosis of transient synovitis to confirmed IgAV.

**Table 2:** Timeline showing the evolution of clinical features, examination findings, investigations, diagnosis, and management throughout the course of illness

Day	Clinical features	Examination findings	Investigations	Impression	Management
Days 1-3	Right knee pain, swelling, and reduced weight-bearing	Afebrile; no rash; localized knee tenderness with reduced range of motion	Knee ultrasound: mild periarticular fluid without joint effusion or collection	Provisional diagnosis of transient synovitis	Analgesia and observation
Day 5	Onset of rash, right elbow involvement, abdominal pain	Erythematous maculopapular rash with subtle areas suggestive of early evolving purpuric change; right elbow swelling; abdomen soft and nontender	Abdominal ultrasound: mesenteric lymphadenitis; no intussusception	Clinicoradiological diagnosis of IgA vasculitis based on evolving multisystem features	Conservative management with monitoring
Day 6	Progression of rash	Increasing lesion density with partial coalescence of lesions	Clinical assessment	IgA vasculitis	Continued conservative management
Day 7	Established purpuric phase	Nonblanching palpable purpura over the lower limbs	Clinical assessment	IgA vasculitis	Observation and follow-up
Days 5-10	Clinically stable	Normal blood pressure (serial); resolving rash and joint symptoms	Serial urinalysis: normal (no hematuria or proteinuria)	No renal involvement detected during the observation period	Follow-up and safety-netting

The patient remained clinically stable with gradual resolution of symptoms. Follow-up evaluation demonstrated improvement in joint symptoms and persistence of resolving purpuric rash. Serial urinalysis remained normal, and blood pressure measurements were within normal limits. However, due to the completion of a planned vacation, the family returned to their home country shortly thereafter. Caregivers were provided with detailed safety-netting advice and recommendations for continued follow-up.

## Discussion

IgAV is the most common systemic vasculitis in childhood and is characterized by immune complex-mediated small-vessel inflammation with IgA deposition [1,2]. The classical clinical tetrad includes palpable purpura, arthritis, abdominal pain, and renal involvement, although these features do not always present simultaneously [1-3].

Musculoskeletal involvement occurs in 70%-80% of cases and is typically transient and nondestructive, most commonly affecting the lower limb joints [2,3]. However, arthritis as an initial and isolated manifestation is less commonly emphasized and may result in early diagnostic uncertainty. Similar presentations, in which cutaneous manifestations follow initial musculoskeletal symptoms, have been described but remain underrecognized in clinical practice [2-4].

This case illustrates a clinically relevant diagnostic challenge: the tendency to anchor on common benign causes of monoarthritis in otherwise well-appearing children. Transient synovitis represents a frequent and appropriate initial working diagnosis in this context. In the present case, the absence of fever, preserved general condition, and ultrasound findings limited to mild periarticular fluid without joint effusion supported this interpretation. The absence of sonographic joint effusion was diagnostically important, as it reduced the likelihood of septic arthritis and supported initial conservative management. At initial presentation, alternative diagnoses such as early IgAV were not apparent, and the diagnostic impression appropriately reflected the most probable explanation based on available findings.

The subsequent clinical evolution, however, illustrates the importance of maintaining diagnostic flexibility. Such presentations underscore the importance of longitudinal clinical observation in pediatric practice, where disease phenotypes may evolve over time. While septic arthritis remains a key differential diagnosis in pediatric monoarthritis, it became progressively less likely in this case given the absence of systemic toxicity, lack of fever, preserved activity level, absence of sonographic joint effusion or collection, and failure to satisfy Kocher criteria for septic arthritis. The emergence of cutaneous and gastrointestinal features shifted the diagnostic probability away from isolated joint pathology toward a systemic inflammatory process.

Although serum sickness-like reaction was considered in the differential diagnosis, given the preceding beta-lactam exposure, the subsequent evolution into characteristic palpable purpura with gastrointestinal involvement favored IgAV.

Gastrointestinal involvement occurs in approximately 50%-75% of cases and ranges from mild abdominal pain to severe complications such as intussusception or gastrointestinal hemorrhage [3,5]. In this patient, mesenteric lymphadenitis without complications was identified, consistent with a mild disease course.

Renal involvement remains the principal determinant of long-term prognosis and may develop later in the disease course [5,6]. Although absent during the observed period, this reinforces the importance of continued monitoring even in clinically mild presentations.

The diagnosis in this case is consistent with the European League Against Rheumatism/Pediatric Rheumatology International Trials Organization/Pediatric Rheumatology European Society classification criteria for IgAV, which require the presence of purpura or petechiae with at least one additional feature, including abdominal pain, arthritis or arthralgia, renal involvement, or histopathological confirmation [7]. The sequential evolution of clinical manifestations, rather than their simultaneous presence, can contribute to early diagnostic uncertainty and necessitate serial clinical reassessment [2-4]. Clinicians should therefore maintain a high index of suspicion and ensure appropriate follow-up in children presenting with unexplained monoarthritis, particularly in the context of recent infection [7,8].

Epidemiological and longitudinal studies further demonstrate variability in disease presentation and outcomes, reinforcing the importance of structured follow-up and risk stratification [9,10]. A key limitation of this report is the absence of longitudinal follow-up. This precludes assessment of delayed renal involvement, a well-recognized complication that may emerge weeks to months after initial presentation, and limits the full characterization of the disease trajectory and long-term outcomes.

Overall, this case reinforces that IgAV should be considered in the differential diagnosis of acute monoarthritis in children and highlights the importance of recognizing the disease as a dynamic clinical entity requiring ongoing reassessment.

## Conclusions

IgAV may present atypically with isolated joint involvement prior to the development of characteristic cutaneous features, creating potential for early diagnostic misclassification as more common benign conditions such as transient synovitis. This case underscores the importance of maintaining diagnostic flexibility in children presenting with acute monoarthritis, particularly in the context of recent infection.

The evolving nature of IgAV necessitates ongoing clinical reassessment as additional systemic features emerge. Early recognition, coupled with appropriate monitoring for renal involvement, remains essential for optimal patient outcomes. Most cases follow a self-limiting course and can be managed conservatively with appropriate safety-netting and follow-up.
